# Comparing the Nutritional Needs of Two Solanaceae and One Cucurbitaceae Species Grown Hydroponically under the Same Cropping Conditions

**DOI:** 10.3390/plants12203642

**Published:** 2023-10-22

**Authors:** Eirini Xaxiri, Evangelos Darivakis, Ioannis Karavidas, Georgia Ntatsi, Dimitrios Savvas

**Affiliations:** Laboratory of Vegetable Production, Department of Crop Science, Agricultural University of Athens, Iera Odos 75, 11855 Athens, Greece; eirini.xaxiri@aua.gr (E.X.); vagos750@gmail.com (E.D.); karavidas@aua.gr (I.K.); ntatsi@aua.gr (G.N.)

**Keywords:** soilless culture, tomato, eggplant, cucumber, uptake concentration, nutrient uptake, floating system

## Abstract

Switching over to closed-loop soilless culture systems, thus preventing pollution of water resources by nitrates and saving water and fertilizers, requires accurate estimations of the mean nutrient-to-water uptake ratios. To contribute to this objective, three fruit vegetable species (tomato, eggplant, cucumber) were grown hydroponically in a floating system under identical cropping conditions to quantify species differences in nutrient uptake. The composition of the nutrient solution used to feed the crops was identical for all species. The total water consumption and the concentrations of most nutrients (K, Ca, Mg, N, P, Fe, Mn, Zn, Cu, B) in the nutrient solution and the plant tissues were measured at crop establishment and at two different crop developmental stages. The obtained data were used to determine the uptake concentrations (UCs) using two mass balance models, one based on nutrient removal from the nutrient solution and a second based on nutrient recovery in the plant tissues. The experiment was conducted in the spring–summer season. The results revealed that the nutrient uptake concentrations were substantially different between species for all nutrients except for N, while there were also significant interactions between the two methods used for their estimation of some nutrients. Thus, the UCs of N, P, Ca, and some micronutrients were significantly higher when its estimation was based on the removal of nutrients from the nutrient solution compared to recovery from plant tissues, presumably because with the first method, losses due to denitrification or precipitation could not be separated from those of plant uptake. The comparison of the three greenhouse vegetables revealed a similar UC for nitrogen, while cucumber generally showed significantly lower UCs for P and for the micronutrients Fe, Zn, and Cu at both cropping stages compared to the two Solanaceae species. The obtained results can be used to precisely adjust the nutrient supply in closed-loop soilless cultivations to the plant uptake thus avoiding both depletion and accumulation of nutrients in the root environment.

## 1. Introduction

The greenhouse production in soilless cropping systems (SCSs) has been dictated by the necessity to improve control over the growth parameters related to the root zone, such as soil-borne pathogen threats, nutrient and water availability in the root environment, salinity, etc. Due to the lack of arable land and the enormous global market demand for vegetables, crops are frequently grown under unfavorable environmental and soil conditions [1]. The cultivation in soilless cropping systems decouples the production from adverse soil conditions such as poor soil fertility, poor soil texture, presence of endemic pathogens in the soil, weeds, salinity, soil temperature, etc. Consequently, an SCS has the potential to render higher yields, increase the produce quality, and extend the areas that can be used for greenhouse cultivation. On the other hand, due to the restricted root volume and the concomitant lower buffering capacity, SCSs are more vulnerable to misguided operations than the crops grown in the soil.

To minimize contamination of aquifers by nutrient emissions originating from fertigation effluents in greenhouse crops and increase the environmental sustainability of protected cultivation, closed-loop SCSs have been developed, henceforth abbreviated as CLSs. In CLSs, the leachates originating from crop fertigation, henceforth termed drainage solution (DS), are collected and recycled. The main environmental advantage of SCSs is the elimination of water pollution by nitrogen and phosphorus emissions contained in the DS. Furthermore, the capture and recycling of the DS restrict the consumption of fertilizers and irrigation water by more than 40% and 30%, respectively [2,3]. However, the CLS requires more knowledge and expertise than the crops in systems with free drainage to ensure a balanced nutrient supply and concomitantly crop productivity. In some northern European countries, such as the Netherlands, CLSs are mandatory to control the contamination of water resources by nutrient emissions [4]. However, in southern European countries, the recycling of fertigation effluents in soilless cropping systems is not frequent. The main difficulties that restrict switching over to CLSs are the possible spread of soil-borne pathogens through the leachates that are captured and reused, and the variable concentrations of nutrients in the recycled DS, which complicate the maintenance of optimal nutrient levels in the root environment of the crops. Furthermore, insufficient competence and expertise of the local advisory services concerning recycling of the fertigation effluents further discourage switching to CLSs.

To support plant nutrition and fertilization and maintain optimal nutrient supply to crops grown in a CLS despite the unpredictable variations in the composition of the DS, suitable decision support systems (DSSs) can be deployed. A DSS should principally be based on suitable nutritional models and algorithms. Innovative DSSs that incorporate the latest state of knowledge can substantially contribute to the restriction of NO_3_ leaching and P runoff to water resources due to inappropriate fertilization practices in agriculture and horticulture [5,6]. Specialty-designed DSSs for CLSs enable precise harmonization between the rates and timing of fertilizer application and the crop nutrient requirements, taking into consideration several variables with an impact on plant nutrition and crop development [3]. However, even the best DSS relies on credible data concerning the nutrient requirements of each cultivated species to successfully support crop nutrition in a CLS.

Solanaceae and Cucurbitaceae are the botanical families with the most important fruiting vegetables cultivated in greenhouses, with tomato and cucumber being the most widespread crop species from each family that are grown under cover. Several investigations have addressed the nutrient requirements of these greenhouse crops when grown in soilless cropping systems [2] and several literature sources provide nutrient recommendations specifically for the cultivation of these crop species in CLSs (e.g., [7,8,9,10,11]). However, the different nutrient solution compositions suggested in these literature sources are based on experiments conducted under different growing conditions. To our best knowledge, there is no study directly comparing the nutrient requirements of these crop species when grown under identical cropping conditions in CLSs. Such studies are important, as they can quantify the differences in nutrient requirements between the compared crop species, thus fully confirming the respective differences in nutrient recommendations provided in the relevant literature sources.

Taking into consideration this gap in knowledge, in the current study we determined the nutrient-to-water uptake ratios by these crops in a floating hydroponic system at two different cropping stages (vegetative/reproductive). The nutrient-to-water uptake ratios, which are commonly termed uptake concentrations (UCs) in the international literature [9], were determined for five macronutrients (N, P, K, Ca, Mg) and four micronutrients (Fe, Mn, Zn, Cu) following two different approaches. The first approach was based on the removal of these nutrients from the nutrient solution, while the second approach was based on the estimation of their recovery from the plant tissues [12]. The aim of the experiment was to identify optimal nutrient management strategies for each crop at each stage of growth and incorporate this information into NUTRISENSE (https://nutrisense.online, accessed on 20 March 2022), which is a decision support system for soilless crops [3]. In the current study, we also included eggplant in the plants under investigation, as the existing knowledge about the nutrient requirements of this Solanaceae species in SCSs, and especially in CLSs, is rather limited [12].

## 2. Results

**Figure 1:**
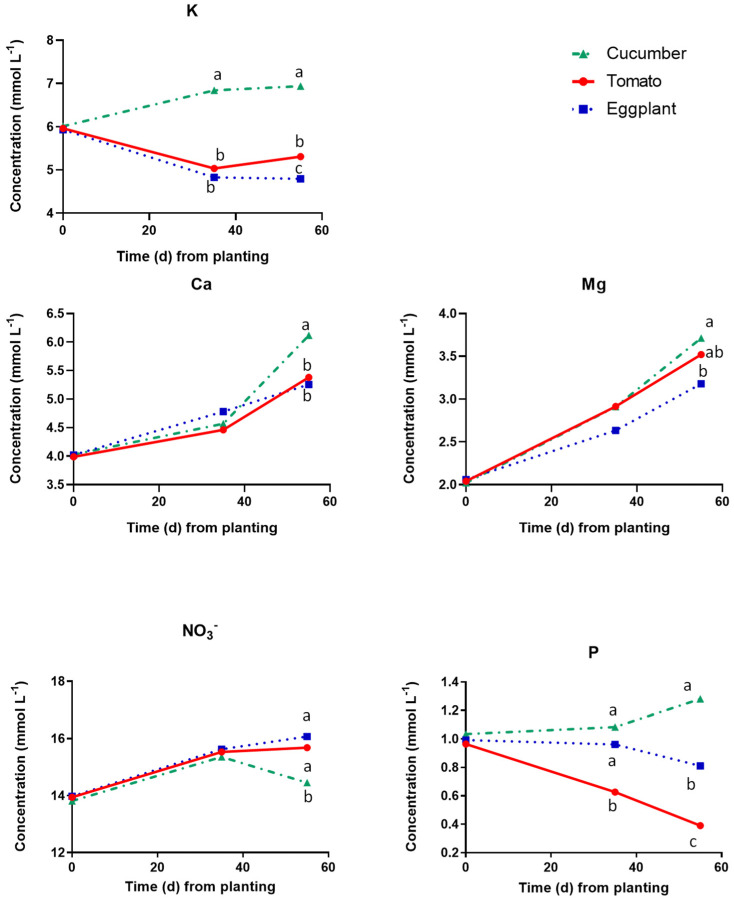
Macronutrient concentrations in the root solution of cucumber, tomato, and eggplant grown in a floating hydroponic system and supplied with a nutrient solution of the same mineral composition at three cropping stages, i.e., at planting (0 DAT), at the end of the vegetative period (35 DAT) and at the end of the experiment, i.e., at full reproductive stage (55 DAT). At each cropping stage, different letters indicate significant differences between the three plant species according to Duncan’s multiple range test (*p* = 0.05).

**Figure 2:**
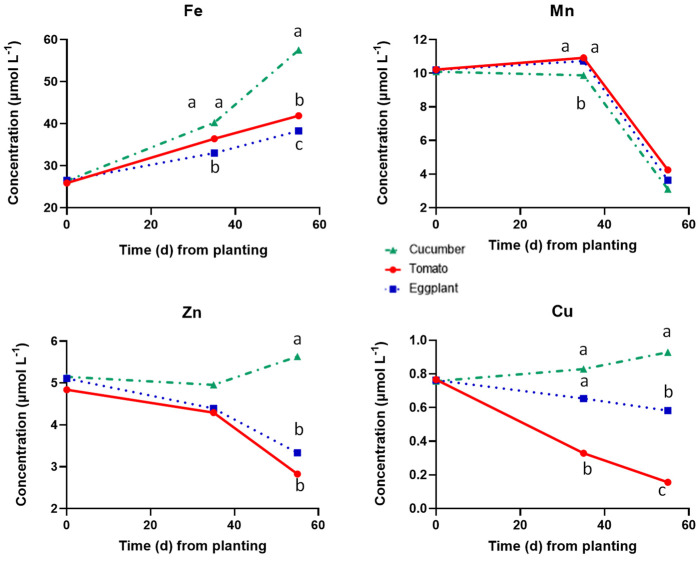
Concentrations of the metallic micronutrients in the root solution of cucumber, tomato, and eggplant grown in a floating hydroponic system and supplied with a nutrient solution of the same mineral composition at three cropping stages, i.e., at planting (0 DAT), at the end of the vegetative period (35 DAT) and at the end of the experiment, i.e., at full reproductive stage (55 DAT). At each cropping stage, different letters indicate significant differences between the three plant species according to Duncan’s multiple range test (*p* = 0.05).

Figure 1 and Figure 2 show the nutrient concentrations measured in the floating tanks (FTs) at three dates during the cropping period. On the 1st sampling date (at planting), the concentrations of all nutrients were similar for all crop species, as the composition of the nutrient solution used to fill the FTs was identical for all of them. However, at the end of the vegetative growing period (35 DAP), substantial differences were observed between the different plant species, indicating commensurate differences in nutrient uptake rates. More specifically, the concentrations of K^+^ increased to appreciably higher levels in the tanks accommodating cucumber, than in those of tomato and eggplant, indicating that their uptake rates by cucumber were lower than those by the two Solanaceae species (Figure 1). The concentrations of NO_3_^−^ as well as those of the bivalent microcations (Ca, Mg) at the end of the vegetative growing stage, i.e., at 35 DAP, were similar in all crop species indicating similar uptake rates. The P concentration in the root zone of cucumber and eggplant did not change up to 35 DAP, while it was reduced in tomato, indicating that this species absorbed P at higher rates than cucumber and eggplant.

During the reproductive stage, the NO_3_^−^ concentration in the FTs with cucumber dropped to lower levels than those in the tomato and eggplant tanks, indicating an appreciable increase in the NO_3_^−^ uptake rates by cucumber after the onset of fruit production (Figure 1). However, the K, Ca, and P concentrations were significantly higher in the tanks with cucumber compared to those with eggplant and tomato during the reproductive stage, indicating substantially lower uptake rates of these nutrients by cucumber during that cropping stage. Finally, the Mg concentration in the tanks with cucumber was slightly but significantly lower than in those used for tomato and eggplant cultivation, indicating higher Mg needs by cucumber during the reproductive stage compared to those of the two Solanaceae species.

At the end of the vegetative period (35 DAP), the concentrations of Mn and Cu were higher in the tanks with cucumber, compared to those measured in the tanks with the two Solanaceae species, while Fe and Zn were similar in all tanks, irrespective of the cultivated species (Figure 2). On the same sampling date, the Fe, Mn, and Zn concentrations were similar in the tanks accommodating the two Solanaceae species, while the Cu concentration was much lower in the tank with tomato compared to that measured in the tanks with eggplant. After the first twenty days of reproductive growth (at 55 DAP), the Fe, Mn, Zn, and Cu concentrations were substantially higher in the tanks accommodating cucumber compared to those measured in the tanks with tomato and eggplant, indicating commensurate differences in uptake rates. The concentrations of Fe, Mn, and Zn were similar in the tanks with the two Solanaceae species at 55 DAP, while that of Cu was significantly lower in the tanks with tomato compared to those with eggplant.

**Figure 3:**
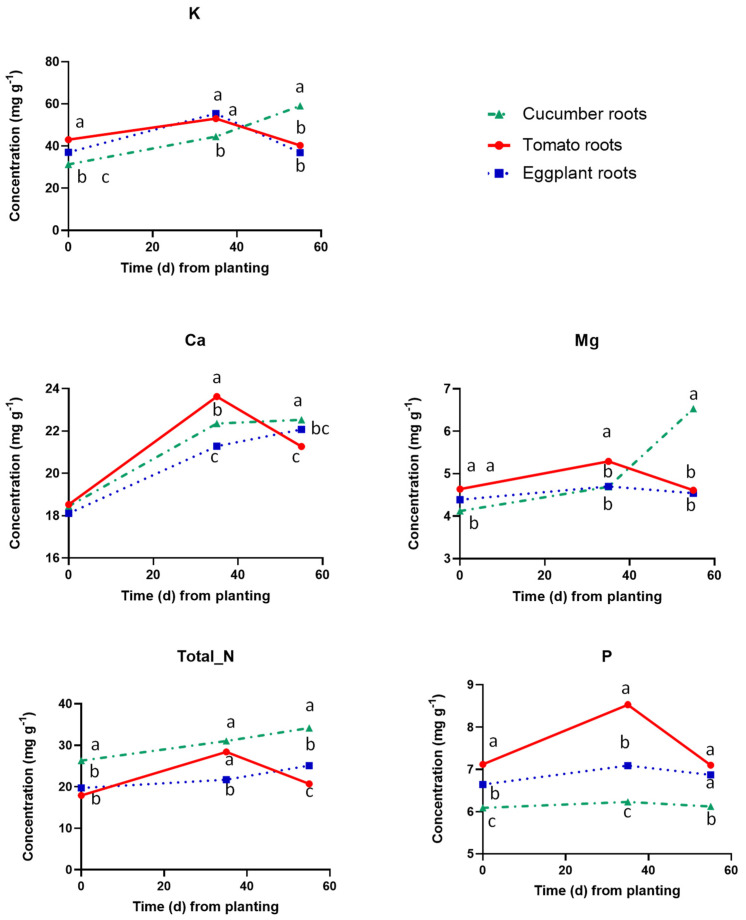
Macronutrient concentrations in dry root tissues of cucumber, tomato and eggplant grown in a floating hydroponic system and supplied with a nutrient solution of the same mineral composition at three cropping stages, i.e., at planting (0 DAT), at the end of the vegetative period (35 DAT) and at the end of the experiment, i.e., at full reproductive stage (55 DAT). At each cropping stage, different letters indicate significant differences between the three plant species according to Duncan’s multiple range test (*p* = 0.05).

Figure 3 shows the concentrations of macronutrients in the root tissues of the three tested fruit vegetables. The total N concentration was substantially higher in the roots of cucumber compared to the two Solanaceae species. Total N exhibited an increasing tendency in the roots of cucumber and eggplant during the reproductive stage, while in tomato, it increased by the onset of the reproductive stage but decreased slightly thereafter. The P concentration in the roots followed a constantly increasing tendency during both the vegetative and reproductive stages, with higher levels in tomatoes at the onset of the reproductive stage, i.e., at 35 DAT. The root K concentration was also similar in all tested fruit vegetables at planting and at the onset of the reproductive stage (35 DAP), with an increasing tendency during that growing period. However, after the transition to the reproductive stage, i.e., at 55 DAP, it decreased substantially in the roots of the two Solanaceae species, while in cucumber roots it increased appreciably. Similarly, with total N, the root Ca concentration followed an increasing tendency in cucumber and eggplant during the whole cropping period, while in tomato, the root Ca reached its maximum by the onset of the reproductive stage but decreased slightly thereafter. At 55 DAP, cucumber exhibited substantially higher root Ca concentrations than the two Solanaceae species. The Mg concentration increased during the vegetative stage in tomato and eggplant but decreased after the transition to the reproductive stage, while in cucumber it was constantly increasing during the whole experimental period.

**Figure 4:**
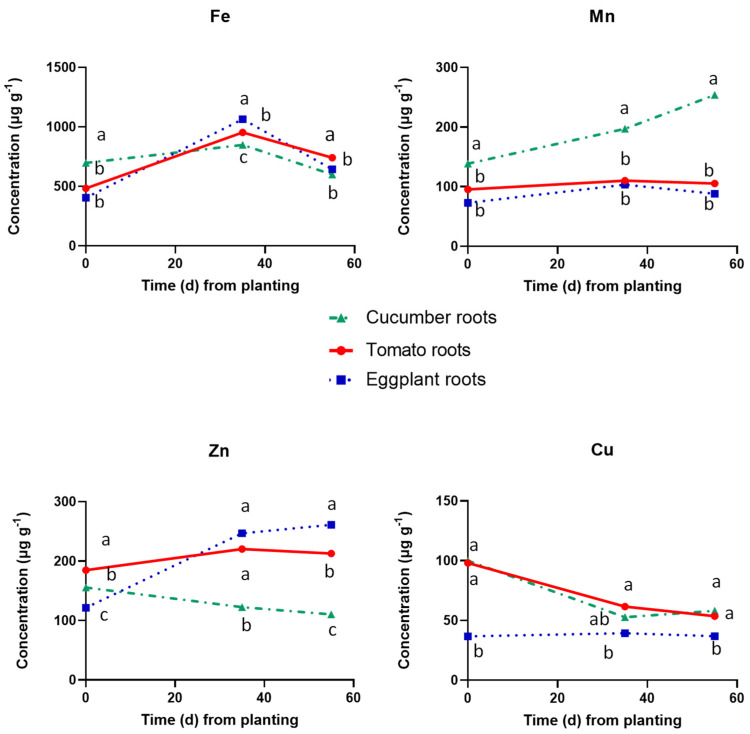
Concentrations of metallic micronutrients in dry root tissues of cucumber, tomato, and eggplant grown in a floating hydroponic system and supplied with a nutrient solution of the same mineral composition at three cropping stages, i.e., at planting (0 DAT), at the end of the vegetative period (35 DAT) and at the end of the experiment, i.e., at full reproductive stage (55 DAT). At each cropping stage, different letters indicate significant differences between the three plant species according to Duncan’s multiple range test (*p* = 0.05).

Figure 4 shows the concentrations of the metallic micronutrients in the roots of the three tested fruit vegetables. The Fe concentration followed an increasing tendency in all three fruit vegetables during the whole cropping period, with higher levels in cucumber at the onset of the reproductive stage (35 DAT) compared to eggplant (Figure 4). The Mn and Zn concentrations also increased at 35 DAP compared to the date of crop establishment in the roots of all three tested vegetables. However, in the roots of cucumber, the Mn and Zn concentrations further increased at 55 DAP, while in the tomato and eggplant roots, they remained at the same level or decreased slightly. Finally, the root Cu concentrations decreased substantially in all three vegetable species at 35 DAP compared to those measured in the seedlings on the date of planting. However, at 55 DAP, the root Cu increased appreciably in cucumber while in eggplant it remained at the same level and in tomato it decreased compared to 35 DAP.

**Figure 5:**
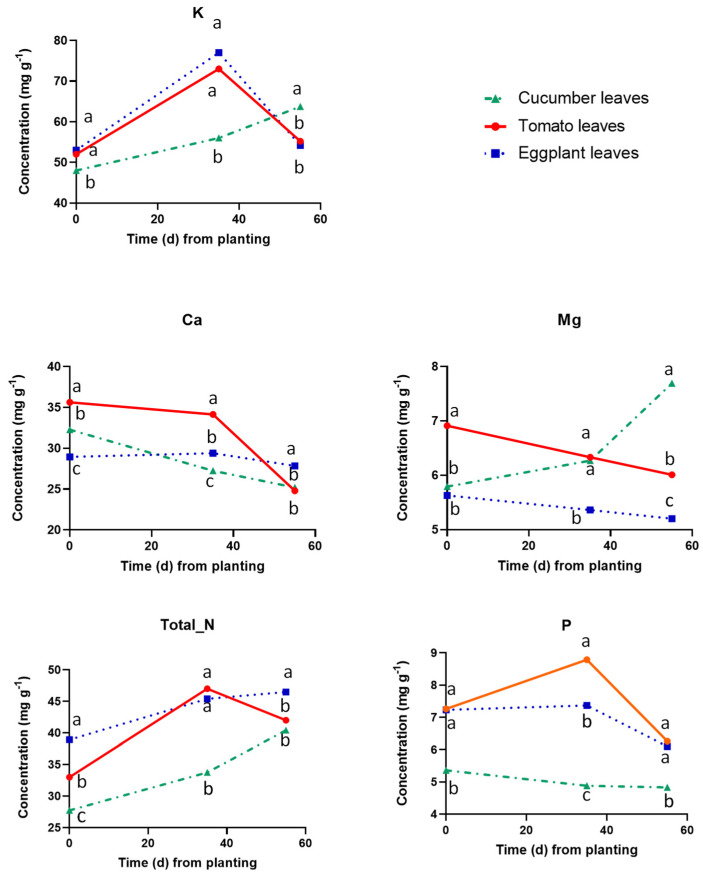
Macronutrient concentrations in dry leaf tissues of cucumber, tomato, and eggplant grown in a floating hydroponic system and supplied with a nutrient solution of the same mineral composition at three cropping stages, i.e., at planting (0 DAT), at the end of the vegetative period (35 DAT) and at the end of the experiment, i.e., at full reproductive stage (55 DAT). At each cropping stage, different letters indicate significant differences between the three plant species according to Duncan’s multiple range test (*p* = 0.05).

Figure 5 shows the concentrations of macronutrients in the leaves of the three tested fruit vegetables. The leaf K, total N, and P levels were similar in the three tested plant species at the onset of the reproductive stage. However, the leaf K and total N concentrations increased at 35 DAP compared to planting, while those of P decreased. At 35 DAP the leaf Ca and Mg were significantly higher in cucumber compared to those found in the two Solanaceae species. At 55 DAT, the leaf concentrations of K, Ca, Mg, and total N were significantly higher in cucumber, compared to tomato and eggplant, while those of P were similar in all plant species.

**Figure 6:**
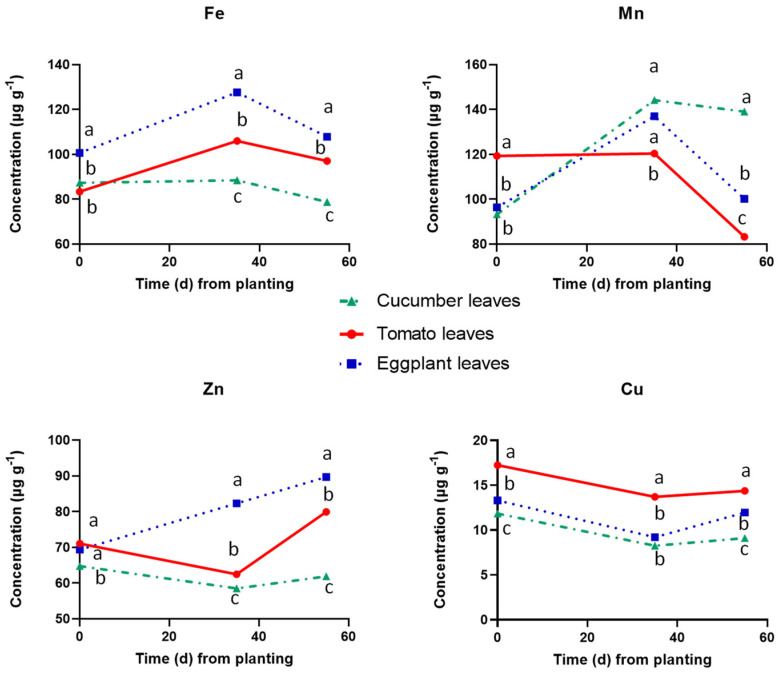
Concentrations of metallic micronutrients in dry leaf tissues of cucumber, tomato, and eggplant grown in a floating hydroponic system and supplied with a nutrient solution of the same mineral composition at three cropping stages, i.e., at planting (0 DAT), at the end of the vegetative period (35 DAT) and at the end of the experiment, i.e., at full reproductive stage (55 DAT). At each cropping stage, different letters indicate significant differences between the three plant species according to Duncan’s multiple range test (*p* = 0.05).

The concentrations of the metallic micronutrients in the leaves of the three tested fruit vegetables are shown in Figure 6. The results show that the leaf Fe, Mn, and Zn concentrations were significantly lower while that of Cu was significantly higher in tomato leaves compared to the other two crop species at 35 DAT. However, at 55 DAT, only Zn was significantly lower in tomato leaves compared to the other two species, while leaf Mn was significantly lower compared only to cucumber and leaf Fe was lowest in eggplant. Overall, the leaf Fe, Mn, and Zn concentrations were significantly higher in cucumber compared to those found in the Solanaceae species at 55 DAT, while leaf Zn was significantly higher also at 35 DAT in cucumber.

The UCs of total N, P, and Ca estimated by considering their removal from the NS during the vegetative period of the three crop species were, for all species, higher than those obtained by considering their recovery from the plant tissues (Table 1). The UCs of K and Mg were similar using both methods of estimation in all crop species. The UCs of total N were similar in all three crop species tested while those of P, K, Ca, and Mg exhibited significant differences. More specifically, the UC of P was highest in tomato, followed by eggplant, while cucumber exhibited the lowest P UC. The UC of K was also lowest in cucumber while the highest value was estimated in the eggplant crop. Eggplant exhibited the lowest Ca UC compared to both tomato and cucumber, and the lowest Mg UC compared only to tomato, while in cucumber the UC of Mg did not differ significantly from those estimated for the other two crop species.

Similarly, with the vegetative stage, during the early reproductive stage (from 35 to 55 DAT) the method of estimation had an impact on the UCs of total N, P, and Mg, but not on those of K and N (Table 2). Furthermore, during the early reproductive stage, the UCs of N and K were similar in all crop species while those of P, Ca, and Mg were different in the three tested vegetable species. More specifically, the UCs of P and Mg were similar in tomato and eggplant, while in cucumber the UC of P was lower and that of Mg was higher compared to the other two crop species. Finally, the UC of Ca was similar at both growth stages in tomato and eggplant, in agreement with that estimated during the vegetative stage. However, the Mg UC in the reproductive stage of eggplant was higher than those estimated for tomato and cucumber, unlike in the vegetative stage where it was lower.

The UCs of the four metallic micronutrients during the vegetative stage were higher when they were determined by considering their removal from the NS than those obtained by considering their recovery from the plant tissues (Table 3). The plant species also influenced the UCs of micronutrients, but its impact was different for each micronutrient. For Fe, the method of estimation showed a significant interaction with the plant species. Thus, while cucumber exhibited significantly lower UCs with both methods of estimation compared to the other two species, and eggplant the highest, the difference between tomato and eggplant was significant only when the estimation was based on plant tissue analysis. Unlike Fe, the other three metallic micronutrients exhibited similar differences in UCs with both methods of estimation. More specifically, cucumber exhibited the highest UC compared to tomato and eggplant, while there was no significant difference between the two Solanaceae species. The Zn UC was highest in eggplant, followed by tomato, while the lowest value was estimated for cucumber. Finally, the highest Cu UC was determined in tomato, followed by eggplant, while cucumber again exhibited the lowest Cu UC.

During the early reproductive stage, the impact of the estimation method on the UCs of Fe, Mn, and Cu was similar to that observed during the vegetative stage, while for Zn a reverse response was observed (Table 4). More specifically, the UCs of Fe, Mn, and Cu were lower, while that of Zn was higher when estimated using data from plant tissue analysis compared to those obtained using NS analysis. The differences in UCs between the three vegetable species during the early reproductive stage followed partly different patterns than those observed during the vegetative stage. A similarity at both developmental stages was that the UCs of Fe, Zn, and Cu for cucumber were considerably lower than those estimated for tomato and eggplant, irrespective of the method of estimation. However, the differences in UCs between tomato and eggplant during the early reproductive stage were not consistent with those estimated during the vegetative stage. Thus, the UC of iron by tomato was slightly higher than that estimated for eggplant with both methods of estimation. The UC of Mn for tomato at the early reproductive stage was similar to that found for eggplant when estimated by considering the plant tissue analysis, while it was lower when estimated by considering the NS analysis. Finally, the UCs of Zn and Cu by tomato and eggplant were similar, irrespective of the method used for their estimation.

## 3. Discussion

Although the water and nutrient needs of plants are governed by fully different physiological processes, their uptake rates are indirectly linked. This happens because the nutrient needs depend on carbon assimilation rates, and carbon uptake shares the same pathways with water uptake, i.e., the stomata aperture [13]. Thus, under standard climatic conditions, when carbon fixation is not limited at the chloroplast level, any factor that determines the whole-plant transpiration rates (e.g., total leaf area, stomata density, whole-plant stomatal conductance) has an analogous effect on total carbon assimilation and thus on nutrient assimilation. Consequently, the ratios of nutrient to water uptake by plants are reasonably stable under standard climatic conditions maintained in acclimated greenhouses [2,9]). Therefore, they are frequently used to establish nutrient solution compositions in soilless culture and especially in closed-loop cropping systems with drainage solution recycling [2,14]. Nevertheless, when climatic factors such as light and air humidity deviate substantially from the standard conditions suggested for greenhouse crops, the transpiration rates fail to relate to carbon assimilation [15]. Consequently, when the climatic conditions deviate from standard levels, the nutrient-to-water uptake rates may deviate substantially from the standard levels. Furthermore, other factors such as cropping conditions and cultural practices that increase or decrease yield, may impose deviations in UCs. Therefore, the UCs suggested in the literature (e.g., [16]) must be used mainly as reference values which should be regularly readjusted during the cropping period based on feedback from the nutrient status in the root zone [3].

The current study showed that the method used has a strong impact on the experimentally estimated UCs of most nutrients. The only UCs not influenced by the method of estimation were those of K and Mg. The method of estimation based on analytical data from the NS resulted consistently in higher UCs at both plant developmental stages and for all nutrients except for Zn, compared to those found when the method was based on analytical results from the plant tissues. This agrees with previous reports concerning determinations of tomato and pepper UCs with these two methods [12,17,18].

The higher UCs of total N, when their estimation was based on nutrient removal from the NS, are presumably due to losses of N from the NS through denitrification, which is a known source of N losses in soilless culture [19]. When estimating UCs by considering the N input/output balance in the NS contained in an FS for a certain time interval, the output also includes the N losses in gaseous form. Thus, the UCs estimated by considering the N concentrations in the NS are apparent UCs since they include the output of the nutrient from the system not only through plant uptake but also through gaseous losses due to denitrification. In contrast, when estimating UCs for the same time interval by considering the N balance in the plant tissues, the obtained values represent only the net nutrient input. Thus, the N UC estimated by considering the N balance in the NS was reasonably higher than that obtained by taking into consideration the N content in the plant tissues. Several investigators have detected substantial losses of N from nutrient solutions used for fertigation in soilless culture which have been estimated up to about 12% of the total supply [19,20,21]). Nevertheless, at high pH levels, the losses of N from NSs due to denitrification may increase substantially. Thus, a pH level of 7 in the root zone of cucumber increased the denitrification-induced N losses from NSs to over 20%, while optimal pH levels ranging between 5.5 and 6.2 could maintain the gaseous N losses below the threshold of 10% [22]. In the current study, the pH in the root zone of all tested vegetable species was maintained consistently below 6.3 and thus the differences observed between the two methods of estimation are consistent with the anticipated losses of N due to denitrification.

Calcium and phosphorus cannot be lost in gaseous forms from NSs, but they can easily form sparingly soluble or insoluble salts that precipitate [23], especially if the pH in the root solution increases to higher levels than the optimal range for plant nutrition [24]. Similarly with N, the losses due to precipitation are included in the UCs of P and Ca when these are estimated by considering the concentrations of these nutrients in the NS. Thus, it is reasonable to estimate higher UCs for P and Ca when the calculations are based on the concentrations of these nutrients in the NS than those estimated by considering their concentrations in the plant tissues.

The cations of the metallic micronutrients (Fe^2+^, Mn^2+^, Zn^2+^, Cu^2+^), when added in an NS, can form insoluble bases of the type M(OH)_2_ (M = Fe^2+^, Mn^2+^, Zn^2+^, Cu^2+^), which lead to losses of these nutrients due to precipitation [25]. The extent of these losses increases with increasing pH in the NS. The pH in the RS/DS can often increase in soilless cultivation [16,24,25] and this can raise the losses of metallic micronutrients in the form of insoluble bases [26]. In the current study, the estimation of higher UCs for the metallic micronutrients when these were calculated using data from chemical analysis of the DS, compared to data from plant tissue analysis, seems to be due to losses through precipitation in the form of M(OH)_2_.

The current study revealed clear differences in nutrient requirements and uptake concentrations between the three tested plant species for most nutrients. Nitrogen was the only nutrient without any significant differences in UCs between the three tested species. However, this was not due to similar N levels in the plant tissues. As shown in Figure 6, the N concentration in the leaves of cucumber was significantly lower than in those of tomato and eggplant, which agrees with the differences obtained when comparing leaf N concentrations reported in the international literature [27,28,29]. However, cucumber produced substantially more plant biomass than tomato and eggplant at the same time interval and, although it also consumed more water, the UCs were similar in all species, despite the lower N concentrations in the plant tissues of cucumber. Unlike that of N, the UC of P in the cucumber crop was significantly lower than those estimated in the two Solanaceae species at both developmental stages. This agrees with the higher P concentrations in the tissues of tomato and eggplant compared to cucumber in the current study. Higher P concentrations in plant tissues of tomato [30] than in cucumber [29] have been reported by other investigators. However, the P levels in the tissues of eggplant [27,31] are not higher than in cucumber. A likely explanation for the substantially lower tissue P concentrations in cucumber is that in water culture systems the uptake of P by cucumber is reduced. This happens because cucumber roots have a higher oxygen demand than Solanaceae [32]. Therefore, cucumber forms a rather weak root system when grown in a pure nutrient solution without an aggregate [33]. However, P is actively absorbed by plants [34] and thus a restricted root system imposes more severe limitations in P uptake than in the uptake of many other nutrients. Cucumber exhibited lower UCs also for Fe, Zn, and Cu, as well as for K at the vegetative developmental stage, which may also have resulted from the lower adaptability of cucumber roots to water culture systems as that used in the current study. Hence, since in the current study the UCs were established in crops grown in a floating system where the oxygen availability is relatively low, they have to be validated also in substrate-grown crops before they can be adopted in cucumber crops grown on substrates.

In completely closed systems with full recycling of the drainage solution, nutrients and water should be supplied at identical ratios to the ratios they are removed from the system [7,35]. Assuming that in a closed soilless crop, the only output of nutrients from the system is the uptake by the plants, the input ratios between nutrient and water, which correspond to their concentrations in the added solution, should be equal to the output ratios, i.e., their uptake concentrations. Consequently, the UC estimated in the current study can be directly suggested as concentrations for nutrient solutions used in closed-loop soilless systems of tomato, eggplant, and cucumber [16]. Nevertheless, as already mentioned, some nitrogen can be removed from a soilless cultivation in gaseous forms due to nitrification, while part of the P, Ca, Fe, Mn, Zn, and Cu can be removed through precipitation after the formation of non-soluble inorganic compounds. These losses, however, must be also replenished when supplying NS in a soilless crop. As already mentioned, these losses of nutrients are included in the UC, when they are estimated using analytical data from nutrient solutions. Therefore, the composition of the added solution used in closed-loop soilless crops should correspond to the UCs estimated by measuring the depletion or accumulation of nutrients in the nutrient solutions and not by measuring nutrient recovery from the plant tissues.

The obtained results can be used to precisely adjust the nutrient supply in closed-loop soilless cultivations to the plant uptake, thus avoiding both a depletion and an accumulation of nutrients in the root environment [3,18]. In addition to their immediate use to compile the composition of nutrient solutions supplied to closed-loop soilless crops, the UCs can also be used to estimate NS compositions for open soilless cultivations. The suggested procedure for a particular crop species is to use a modified version of the concept of accumulation factors as suggested by [7]. According to this concept, the accumulation factor (*R_i_*) for a particular nutrient (*i*) in a certain crop is defined as the ratio between the UC of this nutrient (*C_iu_*), and the optimal concentration of this nutrient in the root zone (*C_ir_*) according to the following equation: *R_i_* = *C_ir_*/*C_iu_* [36]. The *Ri* factors for each nutrient can be determined experimentally for a particular crop species and then used to determine target nutrient concentrations for the supplied solution (*C_is_*), also frequently termed drip solution in substrate-grown crops, by applying the following mass balance equation suggested by Sonneveld (2000) [37]:

(1) $$C_{is}=aC_{ir}+(1-a)C_{iu}$$

where *C_iu_* denotes the UC of the *i* nutrient, *C_ir_* denotes the target concentration of the *i* nutrient in the root zone as estimated using the accumulation factors, and *a* denotes the mean drainage fraction applied (0 ≤ *a* ≤ 1). Using (1), target nutrient concentrations in the supply solution (drip solution) can be estimated as functions of the applied drainage fraction. Following this approach, nutrient solution recommendations for tomato, eggplant, and cucumber can be compiled, which include standard compositions for the added solution, the root solution, and the supplied solution.

## 4. Materials and Methods

### 4.1. Plant Material, Growth Conditions, and Experimental Treatments

The experiment took place in a glasshouse consisting of four compartments at the Agriculture University of Athens (AUA), Greece (37°58′57.9″ N, 23°42′14.3″ E), during the 2022 spring growing season (from 1 April to 25 May 2022). A glasshouse compartment covering an area of 75 m^2^ was used for the current experiment. The experimental plant species were tomato (*Solanum lycopersicum* EMB Belladona GSPP grafted onto HE- MAN A10-0056), eggplant (*Solanum melongena* Beuno A11-25), and cucumber (*Cucumis sativus* Aisopos grafted onto Vitalley E08-0023). Forty plants of each species were used. The tomato and cucumber plants were grafted by the professional nursery Plantas (https://www.plantas.gr/en/, accessed on 1 March 2022).

The seedlings were planted at the stage of 3–4 true leaves into 12 closed-loop hydroponic circuits (experimental plots) on the 1 April 2022. Each circuit was an independent floating hydroponic system consisting of one individual floating tank (FT) filled with nutrient solution (NS) at a depth of 30 cm, 61 cm wide, and 150 cm long, a 50 L tank containing added solution (i.e., NS used to automatically replenish the NS absorbed by the plants in each circuit), a pipe connecting the added solution tank (AST) with the FT, and a floating valve that maintained the NS level in each FT at a constant height of 20 cm from the bottom. The FTs used in the current experiment were constructed from stainless steel by IntelAgro, Thermi, Greece and each of them contained 180 L of NS. The number of plants accommodated in each FT was 10 for all three species and the allocation of the tanks in the glasshouse compartment followed a randomized complete block design, with 4 replicate tanks for each plant species (treatment). Prior to planting, all tanks were filled with NS of the same composition. The composition of the NS used to fill both the FTs and the ASTs (Table 5) was according to Hoagland and Arnon (1950) with small modifications in the concentrations of some micronutrients. The electrical conductivity (EC) of this NS was 2.10 dS m^−1^ and the pH was 5.6. The surface of each tank was covered with perforated styrofoam plates which accommodated the plants and at the same time served to avoid algae growth in the NS. An aquarium pump with an air stone was immersed in each 180 L tank to maintain the dissolved oxygen (O_2_) level at 6 mg L^−1^ in the NS contained in it.

The pH of the NS in the FTs was measured using a portable instrument (Bluelab Combo portable pH, EC, and temperature meter) and adjusted daily or twice per day to 5.60 in all treatments by adding appropriate amounts of 1 N HNO_3_ stock solution. The EC of the NS in the FTs was also measured daily and adjusted to a minimum of 2.10 dS m^−1^ whenever needed by adding concentrated stock solutions of fertilizers. The amounts of HNO_3_ and fertilizer stock solutions added in each individual FT were accurately recorded throughout the experiment. However, there was no adjustment of the NS EC in the FT to an upper threshold level because its level never reached values substantially exceeding the upper thresholds recommended in credible literature sources [9] for the root solution of each species.

### 4.2. Sampling of Nutrient Solutions and Leaf Tissues

Nutrient solution samples were collected from the FTs of all experimental units at planting (1 April), on the 5th of May which was considered the end-day of the vegetative growth stage in all crops, and at crop termination on the 25th of May. After measuring the EC and pH, the collected NS samples were placed into suitable plastic vials and stored at 2 °C until the end of the experiment. Subsequently, they were subjected to chemical analysis to determine the concentrations of N, P, K, Ca, Mg, Fe, Mn, Zn, and Cu.

On the 1st of April, when the plants were planted, 16 randomly selected seedlings of each plant species were sampled and divided into four different samples with four seedlings per sample. The root and the shoots were separately weighed to determine their fresh weight (FW). The samples were placed in an oven at 65 °C (STF-N 400, FALC Instruments S.L.R, Treviglio, Italia) for drying up to constant weight. After 5 days, when the weight of all dried seedlings had been stabilized to a minimum, the samples were weighed to determine their dry weight (DW expressed in gr per plant). Shortly after planting, 2 plants from each of the 12 FTs were randomly selected (4 samples per species, i.e., per treatment) and marked as “sample plant 1” and sample plant 2” for future sampling.

At 35 days after planting (DAP), i.e., on the 5th of May, at the flowering stage of tomato and eggplant and at the start of fruiting in cucumber, the “sample plant 1” was collected from all of the 12 experimental units. The “sample plant 1” was separated into roots, shoots (including flowers and recently set fruit in cucumber), and leaves, and each of these plant parts was weighed and dried at 65 °C to constant weight. Subsequently, the dry weight of each sample was recorded, and all dry tissue samples were stored in a cool and dry place for chemical analysis to determine their nutrient concentrations.

At crop termination on the 25th of May, i.e., 55 DAP, the “sample plants 2” were harvested from all experimental units. The plants were separated again into roots, stems (including flowers), leaves, and fruit and weighed. Subsequently, the collected plant tissues were dried at 65 °C to constant weight and their dry weight was recorded. When the dry weight of all plant parts from all experimental units was stabilized, a sample was collected from each of them, and stored in a cool and dry place for chemical analysis to determine their nutrient concentrations.

Between the second and third sampling periods, all lateral shoots trimmed from the marked plants were collected and their FW and DW were measured. After drying the trimmed lateral shots to constant weight, a sample was collected from each experimental unit and stored for future chemical analysis to determine their nutrient concentrations.

After termination of the experiment, all dried tissue samples were powdered and an amount of 0.5 g from each sample was added into a porcelain cup. The cups with all samples were placed in a chamber furnace LM-112 (Linn High Therm, Hirschbach, Germany) at 550 °C for 8 h until they turned to ash. Subsequently, the ash was extracted using HCl solution (0.25 M) and the extract was filtered through 125 mm Whatman 42 filter paper and put into 100 mL volumetric flasks which were filled up to 100 mL with distilled water. The obtained aqueous solutions were subsequently subjected to chemical analysis to determine the concentrations of N, P, K, Ca, Mg, Fe, Mn, Zn, and Cu.

### 4.3. Determination of Nutrient Concentrations

Nutrient concentration in the NS and the aqueous extracts of plant tissue samples were determined using various methods, depending on the element. The concentrations of Ca, Mg, Fe, Mn, Zn, and Cu in both the NS samples and the aqueous extracts of the plant samples were measured using an Atomic Absorption Spectrophotometer (Shimadzu AA-7000, Kyoto, Japan). The measurements were performed after setting the acetylene gas flow and vacuum pressure in the AA-700 to 1.5 L/min and 3.5 bar, respectively. Potassium was measured by flame photometry in both sample types using a Flame Photometer (Sherwood Model 410, Cambridge, UK). The concentrations of nitrate (NO_3_-N) in the NS samples and phosphorus (P) in both sample types were measured photometrically using a 96-position microplate spectrophotometer (Anthos Zenyth 200; Biochrom, Holliston MA, USA) at 540 nm [38] and 880 nm [39], respectively. The organically bound nitrogen content in the plant tissue samples was measured by applying the Kjeldahl method [40]. The digestion and distillation were performed using a Labtec DT 220 in conjunction with a Scrubber Labtec SR 210 and a Tecator Kjeltec 8200 (FOSS A/S, Hillerod, Denmark). Organically bound N was determined after manually titrating each distilled sample by measuring the volume (mL) of HCl solution (0.05 M) required to change the color of the solution from green to pink. The NO_3_-N concentrations in the aqueous extracts of the plant tissue samples were determined colorimetrically after nitrating salicylic acid and measuring the amount of nitrate at 410 nm using the Anthos Zenyth 200 spectrophotometer. The total N content in the plant tissue samples was obtained by adding the concentrations of organically bound N and NO_3_-N measured in their aqueous extracts.

### 4.4. Estimation of Mean Uptake Concentrations

The uptake concentrations of total N, P, K, Ca, Mg, Fe, Mn, Zn, and Cu were estimated using two different methods to control the credibility of the obtained values and ensure maximum accuracy. The first method was based on measuring the amount of each macro- and micronutrient accumulated in the plants after planting, as estimated in the dry plant tissues, and the recording of the total volume of nutrient solution absorbed by plants in each experimental unit during each of the two growing periods (vegetative and reproductive). More specifically, the mean UCs of total N, P, K, Ca, Mg, Fe, Mn, Zn, and Cu (*C_xu_* in mmol/L for the macronutrients and μmol/L for micronutrients) were estimated using the following mathematical equation suggested by Savvas et al. 2017 [12]:

(2) $$C_{xu}=\frac{C_{xrt}B_{rt}-C_{xrp}B_{rp}+C_{xst}B_{st}-C_{xsp}B_{sp}+\sum C_{xli}B_{li}-C_{xlp}B_{lp}+C_{xft}B_{ft}}{V_{u}}$$

In (2), the subscript *x* denotes the nutrient (*x* = N, P, K, Ca, Mg, Fe, Mn, Zn, Cu), *C* denotes the concentration of the *x* nutrient (in mmol/L or μmol/L) and the subscripts *r*, *s*, *l*, and *f* symbolize roots, shoots, leaves, and fruits, respectively. Furthermore, the remaining indexes *t* and *p* denote the ending and beginning of the growth period, while *i* represents the sampling date sequence of the trimmed lateral shoots and leaves during the experiment. Finally, *B* denotes the dry weight of the plant part (in g/plant) and *V_u_* (L/plant) denotes the cumulative volume of nutrient solution absorbed by plants during each growing period.

The second method used to estimate the UC was based on the computation of the total amount of each nutrient that was absorbed by the plants during each growing period and the recording of the total volume of nutrient solution absorbed by plants in each experimental unit during each of the two growing periods. More specifically, the estimation of the mean UC of the aforementioned nutrients (*C_xu_* in mmol/L for macronutrients and μmol/L for micronutrients) according to the second method was performed using the following mathematical equation suggested by Savvas et al. 2017 [12]:

(3) $$C_{xu}=\frac{V_{r}\left(C_{xb}-C_{xe}\right)+V_{u}C_{xa}}{V_{u}}$$

In (3), *C_xb_* and *C_xe_* (in mmol/L or μmol/L) denote the concentration of the *x* nutrient (*x* = N, P, K, Ca, Mg, Fe, Mn, Zn, Cu) in each FT at the beginning and the termination of each growing period, respectively, while *C_xa_* denotes the concentration of the *x* nutrient in the AS supplied to the crops (Table 1), *V_r_* (in L) denotes the total volume of nutrient solution contained in each FT, and *V_u_* (L/plant) denotes the cumulative volume of nutrient solution absorbed by plants in each experimental unit during each growing period.

### 4.5. Statistical Analysis

The current experiment was set up as a completely randomized design with three treatments corresponding to the three different plant species (tomato, eggplant, and cucumber). To statistically analyze the results, four replicates were set for each plant. The differences in nutrient concentrations found between the three plant species were statistically evaluated by applying one-way ANOVA using the STATISTICA software package, version 12.5 for Windows 9.0 (https://statistica.software.informer.com/9.0/) (Tulsa, OK, USA). However, the mean uptake concentrations were evaluated by applying 2-factorial ANOVA, considering the plant species as one factor and the method of estimation as a second factor. When the ANOVA was significant for a measured parameter, mean separation was performed using Duncan’s multiple range test (*p* ≤ 0.05). In the 2-factorial analysis of the UC data, Duncan’s multiple range test was used to separate the means between the three plant species when only this factor was significant and no interaction between the two factors was found. When the interaction between the two factors was significant according to the ANOVA, the same test was used to separate the means of all six combinations between plant species and the method of estimation.

## 5. Conclusions

The current study revealed that the P, Fe, Zn, and Cu uptake concentrations of cucumber are substantially higher than those of tomato and eggplant, regardless of the plant developmental stage (vegetative or reproductive). Furthermore, the UC of K by cucumber was also lower than by the two Solanaceae species, but only during the vegetative stage. However, since these differences were established in crops grown in a floating system where the oxygen availability is relatively low, and the root system of cucumber is susceptible to low oxygen levels in the root environment, they also have to be validated in substrate-grown crops before they can be adopted in cucumber crops grown on porous media used as substrates. Differences in UC between the two Solanaceae species were observed only for P and Mg in the vegetative stage, which were higher in tomato than in eggplant, and for Ca in the reproductive stage, which were higher in eggplant than in tomato. These differences were consistent with both methods of estimation. However, the method applied to estimate the UC had a strong impact on the results for some nutrients. More specifically, the UCs of N, P, Ca, and some micronutrients were significantly higher when they were estimated based on the removal of nutrients from the nutrient solution compared to recovery from plant tissues. The difference in the UC of N between the two methods is ascribed to losses of N in gaseous forms due to denitrification which could not be separated from the net N uptake when their estimation was based on the removal of NO_3_^−^ and NH_4_^+^ from the nutrient solution. The higher UCs of P, Ca and micronutrients, when their estimation was based on their removal from the nutrient solution, are ascribed to losses through precipitation which also could not be separated from the net plant uptake with that method of determination.

## Figures and Tables

**Table 1:** Uptake concentrations (UCs) of total N, P, K, Ca, and Mg for three different plant species (tomato, eggplant, cucumber), grown in closed hydroponic systems. Values were computed using two different mass balance models fed with analytical results either from plant tissues or from the nutrient solution and refer to the vegetative growth stage (from 1 April to 5 May 2022).

Method of Estimation	Plant Species	Macronutrient UCs (mmol L^−1^)				
		Total N	P	K	Ca	Mg
Plant tissues	Tomato	12.43	1.21	7.16 b	2.75 b	1.12
	Eggplant	11.76	1.02	7.46 ab	2.59 b	0.94
	Cucumber	11.24	0.86	6.37 c	2.85 b	0.98
Nutrient solution	Tomato	14.24	1.36	7.24 b	3.49 a	1.06
	Eggplant	13.63	1.05	7.86 a	2.71 b	1.03
	Cucumber	14.29	0.95	5.41 d	3.46 a	1.10
Main effects						
Method of estimation	Plant tissues	11.81 b	1.03 b	7.00	2.73	1.01
	Nutr. solution	14.05 a	1.12 a	6.84	3.22	1.06
Plant species	Tomato	13.34	1.29 a	7.20	3.12	1.09 a
	Eggplant	12.70	1.04 b	7.66	2.65	0.99 b
	Cucumber	12.77	0.91 c	5.89	3.16	1.04 ab
Statistical significance						
Method of estimation		***	**	NS	***	NS
Plant species		NS	***	***	***	*
Method of estimation × plant species		NS	NS	***	*	NS

In each column, means (*n* = 4) of all treatments when the interaction is significant, or of each main effect when the interaction is insignificant, differ significantly when they are followed by different lower-case letters according to Duncan’s multiple range test. NS: not significant; *, ** and *** indicate significant at *p* ≤ 0.05, *p* ≤ 0.01, and *p* ≤ 0.001, respectively.

**Table 2:** Uptake concentrations (UCs) of total N, P, K, Ca, and Mg during the early reproductive stage (from 1 April to 5 May 2022) for three different plant species (tomato, eggplant, cucumber) grown in closed hydroponic systems. Values were computed using two different mass balance models fed with analytical results either from plant tissues or from the nutrient solution.

Method of Estimation	Plant Species	Macronutrient UCs (mmol L^−1^)				
		Total N	P	K	Ca	Mg
Plant tissues	Tomato	13.37	1.13	6.54	2.56	1.13
	Eggplant	13.14	1.04	6.11	2.89	1.07
	Cucumber	12.68	0.74	6.22	2.40	1.36
Nutrient solution	Tomato	14.82	1.32	5.89	2.76	1.18
	Eggplant	14.33	1.24	6.30	3.23	1.12
	Cucumber	15.75	0.83	6.16	2.68	1.32
Main effects						
Method of estimation	Plant tissues	13.06 b	0.97 b	6.29	2.62 b	1.19
	Nutr. solution	14.97 a	1.13 a	6.12	2.89 a	1.21
Plant species	Tomato	14.10	1.23 a	6.22	2.66 b	1.16 b
	Eggplant	13.74	1.14 a	6.21	3.06 a	1.10 b
	Cucumber	14.22	0.79 b	6.19	2.54 b	1.34 a
Statistical significance						
Method of estimation		***	***	NS	**	NS
Plant species		NS	***	NS	***	***
Method of estimation × plant species		NS	NS	*	NS	NS

In each column, means (*n* = 4) of all treatments when the interaction is significant, or of each main effect when the interaction is insignificant, differ significantly when they are followed by different lower-case letters according to Duncan’s multiple range test. NS: not significant; *, ** and *** indicate significant at *p* ≤ 0.05, *p* ≤ 0.01, and *p* ≤ 0.001, respectively.

**Table 3:** Uptake concentrations (UCs) of the metallic micronutrients for three different plant species (tomato, eggplant, cucumber), grown in closed hydroponic systems. Values were computed using two different mass balance models fed with analytical results either from plant tissues or from the nutrient solution and refer to the vegetative growth stage (from 1 April to 5 May 2022).

Method of Estimation	Plant Species	Micronutrient UCs (mmol L^−1^)			
		Fe	Mn	Zn	Cu
Plant tissues	Tomato	11.78 b	7.51	4.99	1.04
	Eggplant	13.67 a	8.02	6.10	0.78
	Cucumber	10.14 d	8.84	4.25	0.67
Nutrient solution	Tomato	13.62 a	9.24	5.59	1.23
	Eggplant	14.00 a	9.09	6.22	0.94
	Cucumber	11.08 c	10.22	5.20	0.68
Main effects					
Method of estimation	Plant tissues	11.86	8.12 b	5.11 b	0.83 b
	Nutr. solution	12.90	9.52 a	5.67 a	0.95 a
Plant species	Tomato	12.70	8.38 b	5.29 b	1.14 a
	Eggplant	13.84	8.56 b	6.16 a	0.86 b
	Cucumber	10.61	9.53 a	4.73 c	0.68 c
Statistical significance					
Method of estimation		***	***	*	*
Plant species		***	***	***	***
Method of estimation × plant species		***	NS	NS	NS

In each column, means (*n* = 4) of all treatments when the interaction is significant, or of each main effect when the interaction is insignificant, differ significantly when they are followed by different lower-case letters according to Duncan’s multiple range test. NS: not significant; *, and *** indicate significant at *p* ≤ 0.05, and *p* ≤ 0.001, respectively.

**Table 4:** Uptake concentrations (UCs) of the metallic micronutrients for three different plant species (tomato, eggplant, cucumber), grown in closed hydroponic systems. Values were computed using two different mass balance models fed with analytical results either from plant tissues or from the nutrient solution and refer to the early reproductive stage (from 1 April to 5 May 2022).

Method of Estimation	Plant Species	Micronutrient UCs (mmol L^−1^)			
		Fe	Mn	Zn	Cu
Plant tissues	Tomato	12.86 c	7.24 e	7.78 a	0.78
	Eggplant	12.08 d	8.19 e	7.62 a	0.73
	Cucumber	9.35 f	10.17 d	4.24 c	0.60
Nutrient solution	Tomato	17.64 a	18.99 b	6.98 b	0.97
	Eggplant	16.53 b	21.36 a	6.70 b	0.86
	Cucumber	10.26 e	15.78 c	4.42 c	0.66
Main effects					
Method of estimation	Plant tissues	11.43	8.53	6.55	0.70 b
	Nutr. solution	14.81	18.71	6.03	0.83 a
Plant species	Tomato	15.25	13.12	7.38	0.88 a
	Eggplant	14.31	14.78	7.16	0.80 a
	Cucumber	9.81	12.98	4.33	0.63 b
Statistical significance					
Method of estimation		***	***	**	**
Plant species		***	***	***	***
Method of estimation × plant species		***	***	**	NS

In each column, means (*n* = 4) of all treatments when the interaction is significant, or of each main effect when the interaction is insignificant, differ significantly when they are followed by different lower-case letters according to Duncan’s multiple range test. NS: not significant; ** and *** indicate significant at *p* ≤ 0.01 and *p* ≤ 0.001, respectively.

**Table 5:** Nutrient concentrations in the nutrient solution supplied to tomato, eggplant, and cucumber grown in floating hydroponic systems (added solution: AS).

Macronutrient	K	Ca	Mg	NH_4_-N	SO_4_-S	NO_3_-N	P
Concentration (mmol L^−1^)	6.25	4.00	2.00	1.00	1.90	14.00	1.00
Micronutrient	Fe	Mn	Zn	Cu	B	Mo	
Concentration (μmol L^−1^)	25.00	10.00	5.00	0.75	35.00	0.50	

## Data Availability

The data is contained within the manuscript.
